# Application of a strategy based on metabolomics guided promoting blood circulation bioactivity compounds screening of vinegar

**DOI:** 10.1186/s13065-017-0265-5

**Published:** 2017-05-08

**Authors:** Zhangchi Ning, Zhenli Liu, Zhiqian Song, Chun Wang, Yuanyan Liu, Jiahe Gan, Xinling Ma, Aiping Lu

**Affiliations:** 1 Institute of Basic Theory, China Academy of Chinese Medical Sciences, Beijing, China; 2 School of Chinese Materia Medica, Beijing University of Chinese Medicine, Beijing, 100029 China; 3 School of Chinese Medicine, Hong Kong Baptist University, Hong Kong, SAR 00825 China

**Keywords:** Rice vinegar, White vinegar, Metabolomics, Alkaloid metabolites, Promoting blood circulation

## Abstract

**Background:**

Rice vinegar (RV) and white vinegar (WV) as daily flavoring, have also used as accessory in traditional Chinese medicine processing. As we know, the promoting blood circulation efficiency could be enhanced when herbs processed by vinegar. Number of reports focused on health benefits derived by consumption of vinegar. However, few concerned the blood circulation bioactivity.

**Methods:**

In this paper, a metabolomics guided strategy was proposed to elaborate on the chemical constituents’ variation of two kinds of vinegar. GC–MS coupled with multivariate statistical analysis were conducted to analyze the chemical components in RV and WV and discriminate these two kinds of vinegar. The anti-platelet activities in vitro were investigated by whole blood aggregometry platelet test. And the anticoagulant activities were monitored by the whole blood viscosity, plasma viscosity, packed cell volume, prothrombin time, and four coagulation tests (PT, TT, APTT, FIB) in vivo.

**Results:**

Constituents of RV and WV were globally characterized and 33 potential biomarkers were identified. The contents of four potential alkaloid biomarkers increased with aging time prolonged in RV. RV and its alkaloids metabolites exhibited some anti-platelet effects in vitro and anticoagulant activities in vivo. WV failed to exhibit promoting effects.

**Conclusions:**

Alkaloid metabolites were demonstrated to be the principal compounds contributing to discrimination and it increased with aging time prolonged in RV. RV exhibited the blood circulation bioactivity. The alkaloids of RV contributed to the blood circulation bioactivity.

**Graphical abstract:**
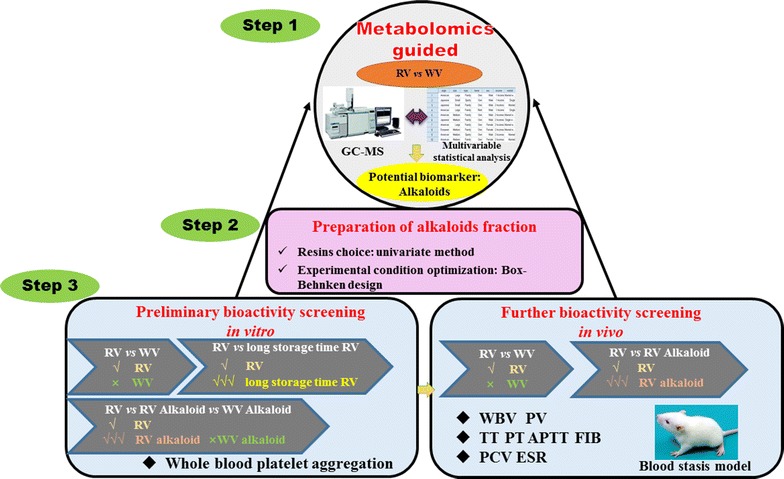
The diagram of metabolomics guided promoting blood circulation bioactivity compounds screening strategy

**Electronic supplementary material:**

The online version of this article (doi:10.1186/s13065-017-0265-5) contains supplementary material, which is available to authorized users.

## Background

Vinegar has been adopted as flavoring dating from around 3000 BC in Asian, European and other traditional cuisines of the world [1]. As evidences accumulated, vinegar was proved to exhibit therapeutic properties, including blood pressure reduction [2], antioxidant activity [2], antibacterial activity [2], reduction in the effects of diabetes [3] and prevention of cardiovascular disease [4]. It is also used as a kind of accessory documented in Lei’s treatise on processing of drugs (LeigongpaozhiLun) (618–907 AD). Numerous Chinese medicines such as Frankincense, Rhizoma Corydalis were believed to enhance the promoting blood circulation therapeutic efficiency after preparation by vinegar [5, 6]. Fruitful researches have been carried on the herbal enhancement of therapeutic efficiency after processing [5, 6], but there are few relative reports concerning the blood circulation bioactivity of vinegar.

Rice vinegar (RV) and white vinegar (WV) are two fermented vinegar, used in China and the United States, produced from rice with distinctive production methods [2]. The production of RV begins with immersion of rice in water, heating, cooling, and inoculation with yeast to produce alcohol [7]. The resultant alcohol was further oxidized to acetic acid by acetic acid bacteria. During aging process, the vinegar aged by insolating in summer and taking out the ice in winter and the flavor components formed. Differently, the WV was fermented from distilled alcohol to acetic acid without aging process.

Vinegar accumulate an overwhelming variety of metabolites that play nondeductible roles in health benefit. During recent years, many studies employed GC–MS technique for quality control and determination of vinegar. Alcohols, organic acids, amino acids, carbohydrates, esters and various micro-constituents were proved to present in vinegar [8]. The previous results showed that the contents of most conventional ingredients (organic acids, free amino acids, carbohydrates) were increased during aging process. Tetramethylpyrazine (TMPZ), a kind of alkaloid metabolites yielding during aging process of vinegar, was used in clinical trials since the 1970s [9]. Reports indicate that TMPZ reduces arterial resistance [10] and increases coronary and cerebral blood flow [10, 11]. A number of alkaloid metabolites are developed as clinical drugs found to have significant biological activities (e.g. berberine and paclitaxel) [12]. Hence variation of alkaloid metabolites should not be overlooked for their exhibit notable function properties.

Since the compositions of vinegar are complicated and partially known, screening bioactive compounds from extracts is a serious challenge. The traditional method is a time-consuming, labor intensive and expensive process, and often leads to loss of activity during the isolation and purification procedures due to dilution effects or decomposition [13]. Through the analysis of metabolites and its variations, metabolomics methods have been established as powerful tools for phenotypes of different production method food [14]. It is well known that GC–MS is widely applied in several analytical fields due to its high sensitive detection for almost both volatile and nonvolatile compounds and its more peak capacity. Many studies showed that the most adopted method is based on GC–MS for the components research of vinegar [8]. The combination of metabolomics and bioactivity screening should fully utilize the power of both techniques, and greatly improve the efficiency of discovery of active compounds.

In our present paper, a strategy based on metabolomics guided bioactivity compounds screening, in which the complex compounds and the synergic effect of multi-targeting were both took into consideration, has been applied in vinegar. GC–MS coupled with multivariate statistical analysis were conducted to analyze the chemical components in RV and WV and discriminate these two kinds of vinegar. The effect of two different vinegars and their alkaloid metabolites on hemorheological disorder were examined by whole blood aggregometry platelet function test in vitro and whole blood viscosity (WBV), plasma viscosity (PV), packed cell volume (PCV), erythrocyte sedimentation rate (ESR), and four coagulation tests (prothrombin time (PT), thrombin time (TT), activated partial thromboplastin time (APTT), fibrinogen (FIB)) in vivo. The aim of this study is to provide scientific information to further understanding the function of vinegar in crude drug processing and its health benefit.

## Methods

### Chemicals

RV from different aging time (1, 4, 5, 7, 14, 20, 30 months) and five batches of WV were collected. The content of TMPZ in different vinegars was determined by HPLC method (Additional file 1: Table S1, Figure S1) [15]. Ion exchange resin (UBK530, WK40, 731, WA30, SK1B) were obtained from Beijing green grass bouquet technology development Co. Ltd. ADP was from Beijing Biotopped Science & Technology Co., Ltd. Arachidonic acid (AA) was purchased from Sigma (St. Louis, MO). TT, PT, APTT, FIB kit was from Beijing Steellex Instrument CO.

### Sample preparation

#### Vinegar chloroform extraction preparation

Vinegar extractions were extracted employing a liquid–liquid extraction process. 1000 mL of vinegar and chloroform were added and extract 3 times. The organic layer was collected and evaporated to dryness. The residue (4.90 g) was stored for the further research.

#### The alkaloid metabolites preparation, qualitative estimation and quantitative evaluation

500 mL vinegar was subjected to 800 mL UBK530 resin column, and eluted with water (fraction A) 3 BV, 50% ethanol (fraction B) 3 BV and 50% ethanol containing 5 M ammonia aqueous 5 BV (fraction C). Fraction C, as the alkaloid fraction, was evaporated to dryness.

Presence of alkaloid was confirmed by Dragendorff’s method [16]. Fraction C was dissolved in HCl and two drops of dragon drops was added. A crystalline precipitate indicates the presence of alkaloid.

The content of total alkaloids in fraction C was determined by the bromothymol blue (BCB) [17]. Accurately measured aliquots (0.4, 0.6, 0.8, 1 and 1.2 mL) of TMPZ standard solution was transferred to different separatory funnels. The absorbance of the complex in chloroform was measured at spectrum of 470 nm in UV-Spectrophotometer against the blank prepared as above but without TMPZ.

### Gas chromatography–mass spectrometry analysis

Gas chromatography–mass spectrometry analysis was performed on GCMS-QP2010 Plus (Shimadzu, Kyoto) equipped with a capillary column (Rxi-50, 30 m × 0.25 mm, 0.25 μm). Helium was used as the carrier gas at a flow rate of 1.0 mL/min. Oven temperature was varied from 60 to 80 °C at 5 °C/min, and then from 80 to 90 °C (3 min held) at 2 °C/min, from 90 to 150 °C (1 min held) at 10 °C/min, from 150 to 220 °C at 1 °C/min, from 220 to 290 °C at 10 °C/min. The injector and interface temperatures were held at 250 °C. Mass spectra in the electron impact mode were generated at 70 eV. The ion source temperature was held at 250 °C. The sample of 1 μL was injected in the split mode injection (split ratio, 60:1). The components were tentatively identified based on linear retention index (RI) and by the comparison of mass spectra with MS data of reference compounds. The linear retention indices were determined for all constituents by using a homologous series of n-alkanes (C_10_–C_40_). The components were identified by comparison of their mass spectra with those of the NIST05 and NIST05S mass spectral library.

### Data processing and multivariate analysis

The number of common components across different samples was selected according to the retention times of the common peaks. Retention times and peak areas for GC–MS was obtained in one table. And then the table was used as input data for multivariate statistic analysis. Multivariate statistical analyses, including unsupervised principal component analysis (PCA) and orthogonal partial least-squares-discriminant analysis (OPLS-DA), were performed using the Simca-P 13.0 statistical package. The critical *p* value for all analyses in this study was set to 0.05. The dataset of selected differential metabolites was imported into MetaboloAnalyst 3.0.

### Animal treatment

Female Sprague–Dawley (SD) rats, weighing 280–300 g, were obtained from the National Institute for Control of Biological and Pharmaceutical Products of China.

After the 30 days administration, the model rats with blood stasis were established by being placed in ice-cold water during the interval between two injections of adrenaline hydrochloride (Adr) and subcutaneously injected with Adr (0.8 mg/kg). After 2 h, the rats were kept in ice-cold water (0–2 °C) for 5 min [18, 19].

### Bioactivity assessment in vitro

Rats were anesthetized with chloral hydrate (300 mg/kg). Blood was drawn from the abdominal aortas to determine. The blood was anticoagulated with heparin (20 U/mL). All platelet aggregation studies were performed using a Chrono-log platelet aggregometer (Chrono-log Co., USA). Single-use cuvettes containing a Teflon-coated stirrer (800 rpm) were filled with pre-warmed 500 μL physiologic saline and 500 μL whole blood. After 10 min of incubation, tests were initiated by adding ADP (10 μM) and AA (0.5 mM). Aggregation was recorded for 6 min.

### Bioactivity assessment in vivo

#### Blood collection

Rats were anesthetized with chloral hydrate (300 mg/kg) 18 h after the last injection of Adr, and blood was drawn from the abdominal aortas to determine. One part of the blood was anticoagulated with heparin (20 U/mL). Another fraction was collected into two plastic tubes with 3.8% sodium citrate (citrate/blood: 1/9, v/v) anticoagulating. Plasma was separated from blood by centrifugation at 3000 rpm for 10 min.

#### Viscosity determination

A total of 1000 μL blood or plasma was used to determine the viscosity with a cone—plate viscometer (Model LG-R-80B, Steellex Co., China) at different shear rates maintained at 37 °C. WBV was measured with shear rates’ varying from 1 to 200/s. PV was measured at high shear rate (200/s) and low shear rate (50/s).

#### ESR and PCV measurements

A total of 1000 μL blood was put into upright westergren tube. The rate of red blood cells falling to the bottom of the tube (mm/h) was observed and reported. The volume of packed red blood cells was immediately measured in the tube after centrifugation (3000 rpm for 30 min).

#### Plasma anticoagulation assay

APTT, TT, PT, and FIB content were examined by a coagulometer (Model LG-PABER-I, Steellex Co., China) with commercial kits following the manufacturer’s instructions.

### Statistical analysis

Data were given as mean ± standard deviation (SD). Multiple comparisons among groups were performed by one-way ANOVA by SPSS Statistics Client 22.0. A value of p < 0.05 was considered statistically significant.

## Results and discussion

### Optimization of GC–MS conditions

Chromatographic parameters such as column type, carrier gas flow, temperature rate, and ion source temperature were adjusted to be able to obtain the best separation for the compounds. The Rxi-50 capillary column obtained the best separation. The carrier gas at flow rate of 1.0 mL/min and the 250 °C ion source temperature were proved to be the most suitable. Established chromatographic conditions and mass spectra conditions are listed in “Gas chromatography–mass spectrometry analysis’’.

### Metabolic profiles of RV and WV

**Fig. 1:**
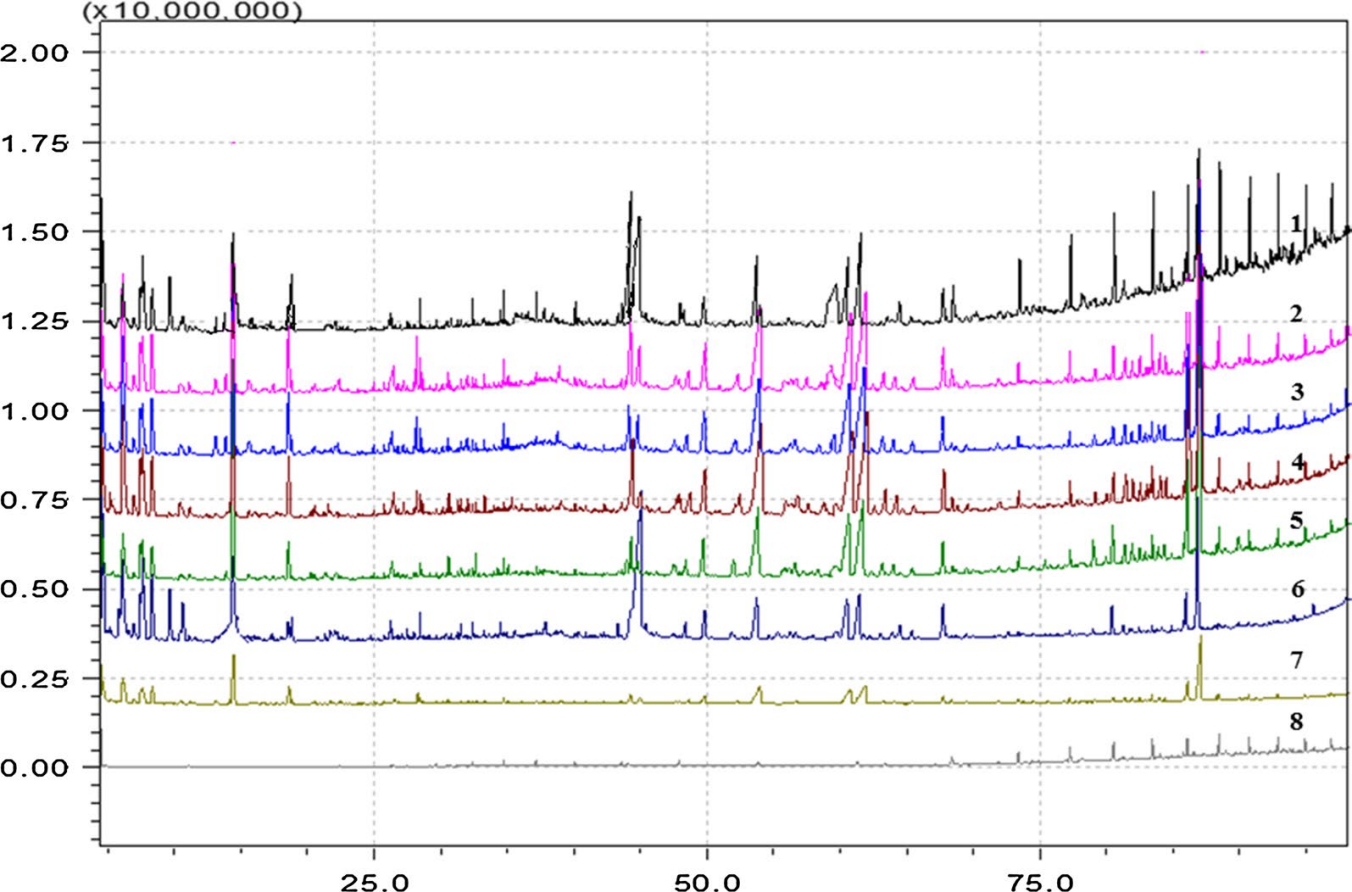
Representative GC–MS chromatography of chloroform extraction of RV and WV (*1* RV with 30 months storage time; *2* RV with 20 months storage time; *3* RV with 14 months storage time; *4* RV with 7 months storage time; *5* RV with 5 months storage time; *6* RV with 4 months storage time; *7* RV with 1 month storage time; *8* WV with 24 months storage time)

Five batches of WV and RV with aging time of 1, 4, 5, 7, 14, 20, 30 months were analyzed. Representative GC–MS fingerprints are presented in Fig. 1. And the compounds in RV and WV are displayed in Table 1. A total of 53 compounds were detected, including different kinds of alcohol, organic acids, amino acids, aldehydes, phenols, ketones, heterocyclics, which were same as those reported in literatures [1, 20].

**Table 1:** Composition of two kinds of vinegar extract

Peak no.	T_R_ (min)	RI	Molecular weight	Molecular formula	Compounds	Index of similarity	VIP	Mean content (%)	
								RV	WV
1	5.83	1022	90	C_4_H_10_O_2_	1,3-Butanediol	93	0.688	0.282	–
2	6.58	1048	192	C_8_H_16_O_5_	6-Deoxy-3-*C*-methyl-2-*O*-methyl	92	0.825	0.302	–
3	6.93	1059	174	C_8_H_14_O_4_	2,3-Butanediol	91	1.047	3.178	–
4	6.93	1059	174	C_8_H_14_O_4_	2,3-Butanedioldiacetate	90	1.028	0.416	–
5	7.46	1083	132	C_6_H_12_O_3_	3-Methoxypropyl acetate	97	1.100	0.866	–
6	7.64	1077	132	C_6_H_12_O_3_	Ethyl 2-hydroxybutyrate	97	1.126	2.128	–
7	8.00	1096	84	C_4_H_8_N_2_	4,5-Dihydro-3-methyl-1H-pyrazole	95	1.097	1.940	–
8	8.37	1106	160	C_8_H_16_O_3_	2-Methoxymethyl-2,4,5-trimethyl-1,3-dioxolane	97	0.905	0.954	–
9	9.64	1151	160	C_7_H_12_O_4_	Trimethylene acetate	94	1.212	1.360	–
10	9.90	1161	102	C_5_H_14_N_2_	Pentamethylenediamine	99	1.224	2.546	–
11	10.37	1176	122	C_7_H_10_N_2_	2,3,5-Trimethyl pyrazine	90	1.250	1.328	–
12	13.97	1251	131	C_6_H_13_NO_2_	Isoleucine	90	1.029	3.088	–
13	14.30	1257	136	C_8_H_12_N_2_	Tetramethylpyrazin	95	1.198	4.772	–
14	15.80	1283	132	C_6_H_12_O_3_	2-Hydrooxy-4-methyl-Pentanoic acid	98	0.620	1.408	–
15	16.16	1289	150	C_9_H_14_N_2_	2,5-Dimethyl-3-isopropylpyrazine	90	0.801	3.398	–
16	16.20	1290	112	C_5_H_4_O_3_	2-Furoic acid	96	0.551	0.078	–
17	17.38	1311	162	C_7_H_14_O_4_	3,4-Dihydroxy-3-methyl-butyl	95	0.894	0.144	–
18	18.52	1331	122	C_8_H_10_O	Phenylethyl alcohol	90	1.063	0.658	–
19	20.18	1360	131	C_5_H_9_NO_3_	2-Acetylaminopropionic acid	86	1.119	2.892	–
20	21.17	1378	85	C_4_H_7_NO	α-Pyrrolidone	93	1.241	1.426	–
21	22.45	1400	286	C_16_H_30_O_4_	2-Ethylhexyl isohexyl ester oxalic acid	91	0.522	1.548	1.312
22	23.00	1415	162	C_6_H_10_O_5_	6-Deoxy-d-mannono-4-lactone	90	0.132	0.152	0.188
23	26.23	1505	137	C_7_H_7_NO_2_	1-Methyl-3-notro-benzene	93	1.230	1.438	–
24	26.46	1511	180	C_9_H_8_O_4_	Phenyl-propanedioic acid	91	0.886	–	0.348
25	26.50	1512	198	C_14_H_3_	2,3,5,8-Tetramethyldecane	94	1.090	–	0.096
26	26.86	1512	342	C_20_H_38_O_4_	Oxalic acid, decyl-2-ethylhexyl ester	95	1.090	–	0.096
27	27.47	1539	146	C_6_H_10_O_4_	Isosorbide	93	0.657	0.198	0.098
28	28.28	1561	126	C_6_H_6_O_3_	5-Butyldihydro-4-methyl-2(3H)-Furanone	93	1.092	0.198	–
29	28.46	1556	150	C_9_H_10_O_2_	4-Hydroxy-3-methoxystyrene	98	0.852	0.610	–
30	28.50	1566	150	C_9_H_10_O_2_	2-Methoxy-4-vinylphenol	92	0.639	0.240	0.074
31	31.50	1672	171	C_8_H_13_NO_3_	*N*-cyclopropylcarbonyl-1-alanine-methyl ester	95	1.247	1.250	–
32	31.98	1690	164	C_10_H_12_O_2_	3-Isopropoxybenzaldehyde	91	0.781	0.132	0.034
33	32.58	1714	146	C_9_H_10_N_2_	3,4-Dimethylpyrrolo[1,2-a]pyrazine	98	1.121	2.980	–
34	33.24	1741	152	C_8_H_8_O_3_	Vanillin	93	0.823	0.308	0.042
35	33.28	1742	152	C_8_H_8_O_3_	4-Hydroxy-3-methoxy-Benzoic acid	98	0.652	0.438	0.152
36	36.72	1874	150	C_9_H_14_N_2_	2,3,5-Trimethyl-6-ethylpyrazine	90	1.154	2.338	–
37	41.13	2027	166	C_9_H_10_O_3_	Hydrocinnamic acid	92	0.520	0.194	0.062
38	43.30	2082	224	C_12_H_16_O_4_	Ethl-β-(4-hydroxy-3-methoxy-phenyl)-propionate	92	0.936	0.459	–
39	45.09	2126	196	C_10_H_12_O_4_	3-(4-Hydroxy-3-methoxyphenyl)propionic acid	90	0.689	5.648	–
40	48.51	2209	170	C_8_H_14_N_2_O_2_	2,5-Dioxo-3-isopropyl-6-methylpiperazine	92	1.005	2.614	–
41	49.84	2229	143	C_7_H_13_NO_2_	3-Pyrrolidin-2-yl-propionic acid	89	1.210	1.674	–
42	54.67	2301	222	C_12_H_14_O_4_	Ethyl(2E)-3-(4-hydroxy-3-methoxyphenyl)-2-propenoate	90	0.359	0.648	0.232
43	56.18	2324	154	C_7_H_10_N_2_O_2_	Hexahydropyrrolo	91	1.233	1.388	–
44	56.23	2324	154	C_7_H_10_N_2_O_2_	1,4-Diaza-2,5-dioxobicyclo	92	1.091	5.494	–
45	58.70	2362	186	C_12_H_14_N_2_	1,2,3,4-Tetrahydro-harmane	90	1.233	4.978	–
46	60.46	2387	210	C_11_H_18_N_2_O_2_	3-Isobutylhexahydropyrrolo	88	1.010	5.046	–
47	61.39	2402	210	C_11_H_18_N_2_O_2_	Leucylprolyl	91	1.083	4.260	–
48	64.20	2449	182	C_12_H_10_N_2_	Harmane	92	1.223	1.832	–
49	67.71	2506	250	C_14_H_22_N_2_O_2_	5,10-Diethoxy-2,3,7,8-tetrahydro-1H,6H-dipyrrolo[1,2-a;1′,2′-d]pyrazine	91	1.245	2.352	–
50	79.12	2761	218	C_12_H_14_N_2_O_2_	3-Benzyl-6-methyl-2,5-piperazinedione	80	1.107	4.068	–
51	81.40	2832	246	C_14_H_18_N_2_O_2_	2-Benzyl-3,6-dioxo-5-isopropylpiperazine	81	1.193	1.868	–
52	86.80	3036	583	C_33_H_37_N_5_O_5_	Dihydroergotamine	86	1.094	5.458	–
53	87.11	3047	244	C_14_H_16_N_2_O_2_	3-Benzylhexahydroprrolo[1,2-a]pyrazine-1,4-dione	92	1.003	2.742	–

**Fig. 2:**
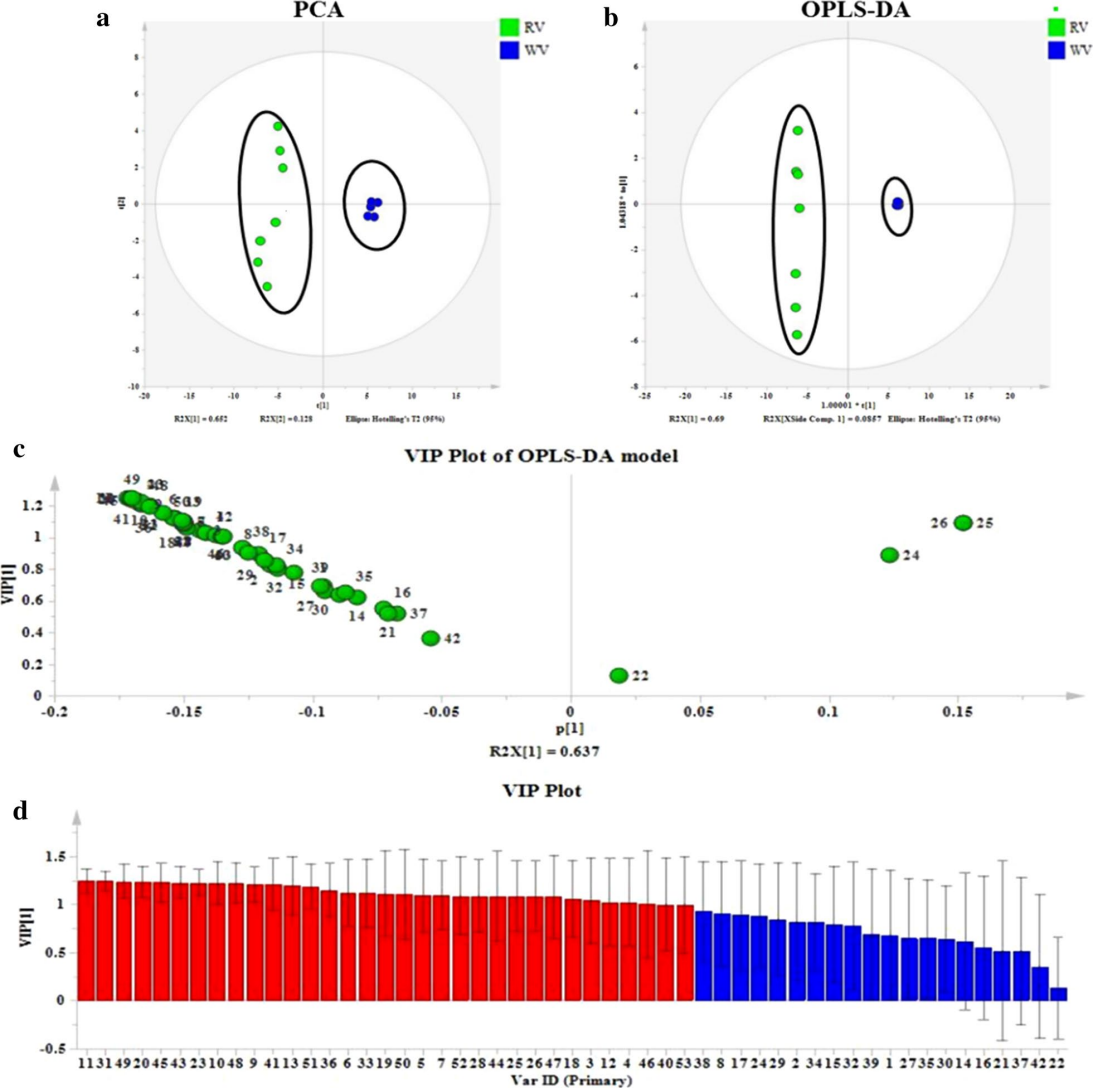
Multivariate statistical analysis of RV and WV. **a** PCA score plots of two different kinds of vinegar; **b** OPLS-DA of two different kinds of vinegar; **c** variable important (VIP) plot of OPLA-DA model between two different kinds of vinegar; **d** VIP plot of two different kinds of vinegar

PCA and OPLS-DA were utilized to classify the metabolic phenotypes and identify the differentiating metabolites. A PCA score plot for first and second principal components was utilized to depict the general variation among the samples of two dosage forms (R^2^X = 0.78, Q^2^ = 0.987). The PCA scores plot could divide the different samples into separate blocks, suggesting that the different samples into distinguish two kinds of vinegar (Fig. 2a). OPLS-DA was employed for classification or discrimination analyses. A loading plot predicates the list of metabolites helping in the positioning of the distance from diverse groups. Metabolic markers of RV and WV were plotted by the OPLS-DA, depicting the variable metabolic patterns at the phenotype (Fig. 2b). A VIP plot was used to identify the metabolites according to the orders of their contributions to the separation of clustering (Fig. 2c). The farther away from the origin, the higher value of the ions in VIP scores plot. Potential markers were extracted from VIP plots constructed following the OPLS analysis, and markers were chosen based on their contribution to the variation and correlation within the dataset (Fig. 2d). The predictive ability Q^2^Y of 0.997 was obtained.

### Potential biomarker between RV and WV

#### Characterization, Bioactivity retrieving and validation

VIP values reflected the overall importance of the variables in the model. Variables with a larger VIP are more relevant for sample classification. The VIP plot (Fig. 2c, d), was used to assist in finding the most relevant variables which contributed to distinguish between two different kinds of vinegar. 33 metabolites were identified and selected as potential biomarkers (as shown in Table 1) .24 of them were all belonging to the alkaloid metabolites. In the present study, the compounds in vinegar were identified using their mass spectra, RI, authentic compounds, and were compared with respect to their relatively quantitative characteristics. Information on the chemical components of the vinegar is useful and necessary for the further study.

The bioactivities of potential biomarkers were obtained via PubChem (http://www.nvbi.nlm.nih.gov/pccompound) and Scifinder. TMPZ, Dihydroergotamine, harmine and 1, 2, 3, 4-tetrahydroharmine were screened and verified (as shown in Additional file 1: Figure S2). Harmine and 1, 2, 3, 4-tetrahydroharmine possess antiplatelet activity and vessel expansion activity. Acetylcholinesterase inhibitory activity is one of the proposed targets for indole analogs. Harmane, a β-carboline structure with 1-methyl substituted, displayed a good inhibitory activity on acetylcholinesterase with inhibition more than 80%. The tetrahydro-β-carboline analog showed a tendency to reduce the inhibitory activity compared to the other less flexible b-carboline [21]. Dihydroergotamine is 5-HT receptor agonists, and two of the most widely used drugs for the acute treatment of migraine attacks [22]. Ergotamine was infamous in former centuries for causing ergotism and miscarriages when ingested through infected bread [23]. Dihydroergotamine is derived from ergotamine are both constrictors of cranial arteries. It is less potent in constricting peripheral arteries than ergotamine, but is more potent in constricting peripheral veins [24]. The results showed that they were only can be detected in RV.

A two-stage ROC curve analysis was applied to validate the potential biomarkers. The area under the ROC curve is a summary measure that essentially averages diagnostic [25]. The four potential biomarkers with the areas under the ROC curves were 1, which considered to show the diagnostic accuracy (as shown in Additional file 1: Figure S3).

#### Trends of time series analysis of 4 potential biomarkers in RV from different aging time

As elaborated in “Characterization, bioactivity retrieving and validation’’, four potential biomarkers can be only detected in RV. So changes of four potential biomarkers, during the aging process of the final product of RV were tested next. C_22_ was selected as a reference substance. Relative peak area of four potential biomarkers was calculated by the ratio of their peak area to C_22_ peak area (Additional file 1: Table S2). After stored for 30 months, relative peak areas of four compounds was increased. The results suggested that their contents increased with aging time.

A time series is a series of data points listed (or graphed) in time order. Time series analysis comprises methods for analyzing time series data in order to extract meaningful statistics and other characteristics of the data [26]. We analyzed seven time-series (1, 4, 5, 7, 14, 20, 30 months) from RV samples. Trend images of the four potential biomarkers (Additional file 1: Figure S4) showed that the contents of them increased with aging time and the trends over time for the content were linear. Mean absolute percentage error (MAPE) showed a good ability for discriminating time series trend of these four potential biomarkers. Mean absolute deviation (MAD) and mean squared deviation (MSD) are believed to be the discrimination of the model accuracy. The value of MAD and MSD reflected the accuracy of time series trend.

Raw vinegar was steam cooked, sealed in ceramic containers, and stored outdoors for months or longer in order to accelerate the synthesis of abundant aromatic and functional materials, such as esters and TMPZ [1]. Changes of aromatic and functional materials in aging process were learned in recent years. It is suggested that the content increase of TMPZ during vinegar aging was primarily due to the Maillard reaction [1, 20]. The product mechanism of the other potential biomarkers needs to further investigate.

### Alkaloids preparation, qualitative and quantitative estimation

#### Optimization of column chromatographic separation conditions

According to the guide of metabolomics research, alkaloid compounds were proved to be the main characteristic markers in two kinds of vinegars. The column chromatography was developed to isolate the alkaloid part from the 30 months-aging-time RV for the further bioactivity study.

The use of a suitable column packing represents one of the most critical choices of the entire separation procedure. Static absorption of five ion exchange resins was evaluated by univariate method. An overall evaluation of data showed that the larger loading capacity, and less irreversible adsorption was clearly obtained performing analysis with UBK530.

The elution solvent, the volume of vinegar and the volume of resin and elution rate have been taken in consideration as variables. In order to optimize the preparation parameters, a Box-Behnken design (BBD) was conducted (Additional file 1: Figure S5). The four factors were designated and prescribed into three levels (as shown in Additional file 1: Table S3). All experiments were performed in triplicate and the averages of total alkaloid content were taken as response.

#### Qualitative estimation and content determination of total alkaloids

Fraction C showed positive alkaloid during the qualitative estimation assay by Dragendorff’s method as described in “The alkaloid metabolites preparation, qualitative estimation and quantitative evaluation’’. A yellow colored complex with a maximum absorption was developed. The content of total alkaloids in fraction C was 64.82 mg/g. And fraction C was injected for GC–MS analysis for the qualitative and quantitative validation.

### Bioactivity assessments of two kinds of vinegars

#### Validation of promoting blood circulation activity of vinegars in vitro

**Fig. 3:**
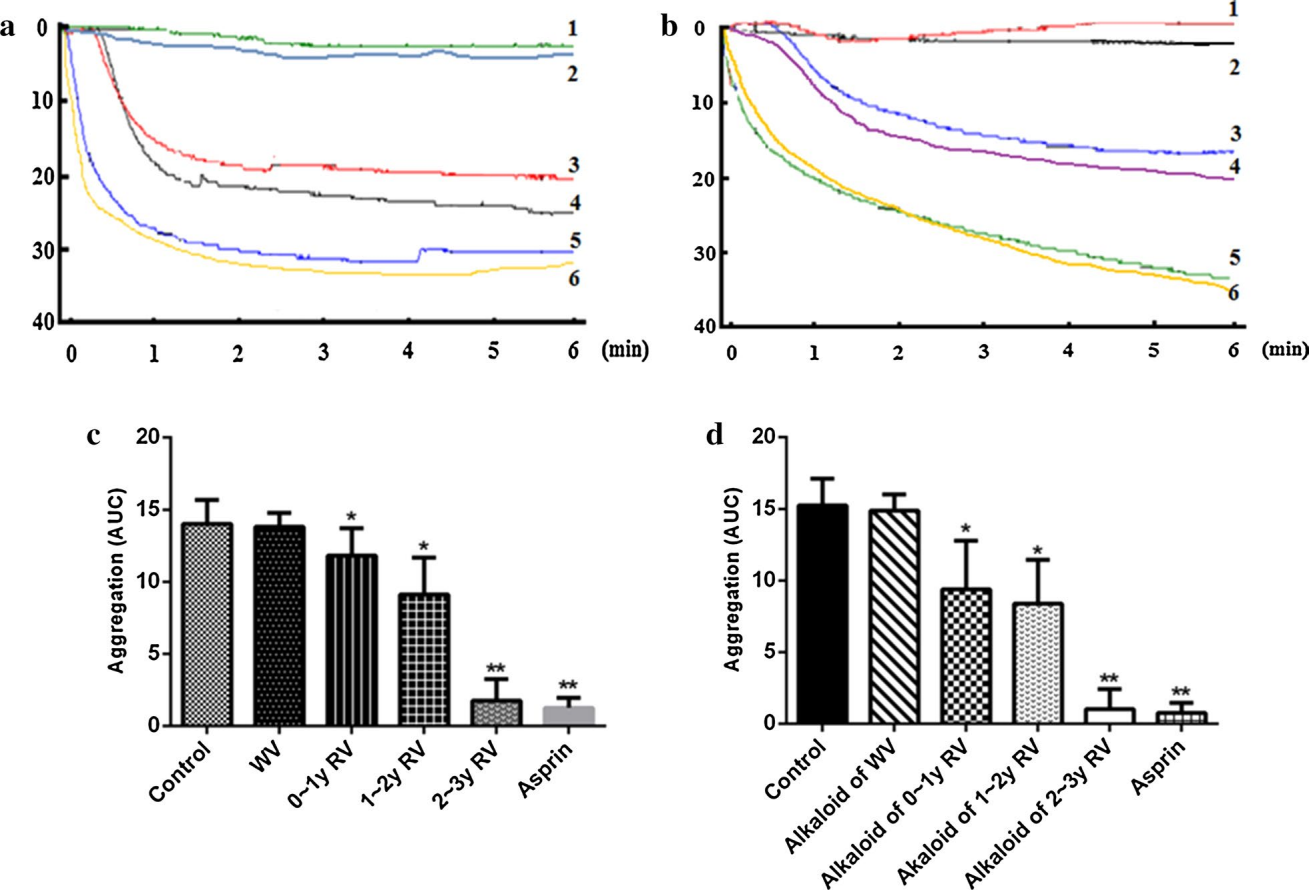
**a** Effects of RV and WV on antiplatelet in vitro (*1* Aspirin group; *2* 2–3 year aging processed RV group; *3* 1–2 year aging processed RV group; *4* 0–1 year aging processed RV group; *5* WV group; *6* Control group); **b** alkaloids metabolites of different vinegar inhibition of AA induced platelet aggregation in vitro (*1* Aspirin group; *2* Alkaloids metabolites of 2–3 year aging processed RV; *3* Alkaloids metabolites of 1–2 year aging processed RV; *4* Alkaloids metabolites of 0–1 year aging processed RV; *5* Alkaloids metabolites of WV; *6* Control group); **c** AUC value of different vinegars inhibition of ADP induced platelet aggregation in vitro; **d** AUC value of alkaloids metabolites of different vinegar inhibition of AA induced platelet aggregation in vitro. ‘*’ and ‘**’, p < 0.05 and p < 0.01 respectively, comparison with the normal control group

Platelet aggregation is thought to be one of the factors that determine blood viscosity [27]. Results of ADP-induced aggregometry measured in whole blood are presented in Fig. 3a, c. The positive control, aspirin, significantly decreased the platelet aggregation. Interestingly, RV produced an aging time -dependent anti-platelet effect (as shown in Fig. 3a). Treatment with 2–3 years aging process RV could markedly decrease. While WV failed to show an anti-platelet effect. The result indicated that long aging time could enhance the quality of the vinegar and greatly improves its health-care function.

Results of AA-induced aggregometry measured in whole blood are presented in Fig. 3b, d. Against AA-induced platelet aggregation responses, the test could successfully demonstrate the anti-platelet effect of alkaloid metabolites of RV with different aging time. Treatment with 1–2 years or 0–1 year aging process RV alkaloid metabolites could also significantly decrease but with less potent in comparison with 2–3 years aging process RV alkaloid metabolites. The alkaloid metabolites of WV also failed to show an anti-platelet effect. We found that the dissociation of the carboxyl of AA was restrained in the acidic medium, which made it impossible to induce the platelet aggregation.

#### Validation of the promoting blood circulation activity of vinegars in vivo

Following the results of the anti-platelet research, the RV with 30 months of aging time was employed as experimental material. The effects of vinegar chloroform extraction and alkaloid extraction in vivo were shown in Table 2. WBV is the reflection of intrinsic resistance of blood to flow in vessels [28]. And PV could reflect the type and concentration of the proteins in plasma to a certain extent [18]. The alkaloid metabolites of RV remarkably decreases PV and WBV at all shear rates (p < 0.01). The PV of RV at different concentration groups significantly decreased compared with the model group (p < 0.05). And the WBV at different shear rates in the blood stasis were partially deduced by different concentration RV groups. They were also effective in decreasing ESR and PCV. However, the WV group showed no significant downward trend. PT, APTT and TT reflect the activity of the extrinsic, intrinsic and both pathways of coagulation and thus are parameters of the anticoagulation state of the plasma [29, 30]. PT is used to evaluate the overall efficiency of the extrinsic clotting pathway. A prolonged PT indicates a deficiency in coagulation factors V, VII, X. On the other hand, APTT is a test of the intrinsic clotting activity [31]. In alkaloid metabolites group, alkaloid metabolites of RV and RV groups significantly prolonged TT and APTT, increased PT and decreased FIB content. WV group had no effects on plasma coagulation parameters. The equivalent amount of TMPZ of RV was not affected.

**Table 2:** Valid the promoting blood circulation activity of vinegars in vivo

Groups	WBV					PV	TT (s)	PT (s)	FIB (g L^−1^)	APTT (s)	ESR	PCV
	1	3	30	100	200							
N	11.06 ± 1.12	8.18 ± 1.15	4.69 ± 0.25	3.63 ± 0.19	3.16 ± 0.15	1.45 ± 0.06	28.77 ± 4.20	9.80 ± 0.30	1.76 ± 0.22	12.93 ± 0.94	0.62 ± 0.75	44.10 ± 3.17
M	23.52 ± 3.12**	13.51 ± 1.60**	6.25 ± 0.45**	4.76 ± 0.50**	3.85 ± 0.24**	1.66 ± 0.07**	24.60 ± 1.22*	8.50 ± 0.37**	5.59 ± 0.45**	10.07 ± 0.72**	2.83 ± 2.32**	51.87 ± 5.53**
W	28.74 ± 2.01	15.93 ± 0.96	6.23 ± 0.88	5.23 ± 0.20	4.18 ± 0.16	1.64 ± 0.03	36.88 ± 2.83^##^	8.92 ± 0.25	5.58 ± 0.44	14.26 ± 0.55^#^	1.72 ± 1.23	48.48 ± 2.85
RL	23.83 ± 2.85	13.30 ± 1.03	6.07 ± 0.33	4.48 ± 0.38	3.77 ± 0.17	1.52 ± 0.07^##^	40.88 ± 3.78^##^	9.15 ± 0.33^##^	5.41 ± 0.45	14.37 ± 1.07^#^	1.80 ± 1.15	50.66 ± 2.01
RH	23.70 ± 2.69	13.71 ± 1.14	6.10 ± 0.19	4.52 ± 0.32	3.78 ± 0.14	1.51 ± 0.06^##^	37.72 ± 3.45^##^	9.66 ± 0.35^##^	4.64 ± 0.32^##^	15.68 ± 1.41^##^	1.30 ± 1.12^#^	52.07 ± 3.09
AER	18.71 ± 2.54^##^	11.69 ± 1.52^#^	5.72 ± 0.35^#^	4.16 ± 0.22^##^	3.54 ± 0.20^##^	1.51 ± 0.05^##^	39.37 ± 4.98^##^	9.43 ± 0.51^##^	5.26 ± 0.66^#^	13.55 ± 1.29^#^	0.63 ± 0.73^##^	45.42 ± 3.22^##^
TMPZ	23.24 ± 2.80	13.57 ± 1.25	6.03 ± 0.42	4.47 ± 0.49	3.74 ± 0.21	1.57 ± 0.05^#^	35.12 ± 4.13^##^	9.02 ± 0.25^#^	5.54 ± 0.83	15.45 ± 0.55	1.00 ± 0.88^#^	47.98 ± 2.02
Asp	18.89 ± 1.70^#^	12.51 ± 1.27^#^	5.86 ± 0.26^#^	4.13 ± 0.07^##^	3.52 ± 0.10^##^	1.53 ± 0.10^#^	38.20 ± 2.01^##^	9.35 ± 0.44^##^	4.68 ± 0.34^##^	15.37 ± 0.81^#^	0.58 ± 0.88^##^	46.72 ± 3.93^##^

Data represent mean ± SD n = 8

*N* Normal group, *M* Model group, *W* WV group, *RL* RV low dosage group, *RH* RV high dosage group, *AER0* Alkaloid extraction of RV

* p < 0.05 vs. control group

** p < 0.01 vs. control group

^#^p < 0.05 vs. model group

^##^p < 0.01 vs. model group

Tests in vivo further indicated that RV and its alkaloid metabolites possess promoting blood circulation activity. The results may also create valuable insight into the possible effects and utilization of vinegar and its alkaloid metabolites as nutrition. Although RV and its alkaloid metabolites could improve the blood fluidity, the equivalent amount of TMPZ in RV failed to show the bioactivity of promoting blood circulation. It was surmised that some ingredients in RV could enhance the promoting blood circulation activity.

### Strategy

Strategy based on metabolomics guided bioactivity compounds screening includes the following steps. First, GC–MS was conducted to analyze the chemical constituents in RV and WV. Alkaloid metabolites were proved to be the principal potential biomarkers. TMPZ, dihydroergotamine, harmine and 1,2,3,4-tetrahydroharmine were screened as potential biomarkers possessed promoting blood circulation bioactivities. And the contents of them increased with aging time in RV. Second, the alkaloid metabolites were isolated. Third, the test of anti-platelet was conducted to validate the promoting blood circulation activity of WV and RV with different aging time preliminarily. Finally, the promoting blood circulation activity study in vivo was carried out. Anticoagulant activities were examined by monitoring the WBV, PV, ESR, PCV, and four coagulation tests.

## Conclusions

In this work, a strategy of bioactivity compounds screening based on metabolomic guided was established. The chemical analysis and multivariate statistical analysis were conducted for classification of RV and WV. Constituents of RV and WV were globally characterized by GC–MS and 33 potential biomarkers were identified. Alkaloid metabolites were proved to be the main compounds contributing to discrimination of two kinds of vinegar and verified only in RV. TMPZ, dihydroergotamine, harmine and 1,2,3,4-tetrahydroharmine were screened and the contents of the four potential biomarkers increased with aging time by semi-quantitative analysis and trends of time-series analysis. With the guidance of metabolomics research, alkaloid metabolites were isolated. The anti-platelet in vitro confirmed an effect of RV and its alkaloids metabolites preliminarily. RV and its alkaloids metabolites further were endowed with in vivo by monitoring WBV, PV, ESR, PCV, and four coagulation tests. WV failed to exhibit the effect of promoting blood circulation. Both the tests of bioactivity in vitro and in vivo are validated the results of metabolomics research. Promoting blood circulation activity of RV may make it to assist the several promoting blood circulation therapeutic efficiency of traditional Chinese medicines after processing. Compared with the traditional isolation and purification method, the established strategy combined of metabolomics and bioactivity screening we proposed should fully utilize the power of both techniques, and greatly improve the efficiency of discovery of active compounds.

## Additional file

**Additional file 1: Table S1.** The content of TMPZ in RV and WV. **Table S2.** The peak area and the relative peak area value of four potential biomarkers in different aging period. **Table S3.** The levels and factors investigated in BBD. **Figure S1.** HPLC chromatogram of TMPZ. **Figure S2.** The results of bioactivity screening. **Figure S3.** Diagnostic efficacy evaluation using ROC curves of the four potential biomarker metabolites in two different vinegar. **Figure S4.** Trends of time-series analysis graphs of four potential biomarkers. (A) TMPZ (MAPE: 2.05853, MAD: 1.67627, fitted curve: Yt = 60.81+5.089xt); (B) Dihydroergotamine (MAPE: 1.63096, MAD: 0.15345, fitted curve: Yt = 6.726+0.7121xt); (C) Harmine (MAPE: 1.72704, MAD: 0.01711, fitted curve: Yt = 0.7764+0.05780xt); (D) 1,2,3,4-tetrahydroharmine (MAPE: 3.76071, MAD: 0.04998, fitted curve: Yt = 0.9695+0.0910xt). **Figure S5.** Response surfaces estimated from the full factorial design for the content of total alkaloids.

## Abbreviations

RV: rice vinegar
WV: white vinegar
TMPZ: tetramethylpyrazine
WBV: whole blood viscosity
PV: plasma viscosity
ESR: erythrocyte sedimentation rate
PCV: packed cell volume
AA: arachidonic acid
BCB: bromothymol blue
RI: retention index
PCA: principal component analysis
OPLS-DA: orthogonal partial least-squares-discriminant analysis
VIP: variable influence on projection
AUC: area under curve
ROS: receiver operating characteristic curves
APTT: activated partial thromboplastin time
TT: thrombin time
PT: prothrombin time
FIB: fibrinogen
MAPE: mean absolute percentage error
MAD: mean absolute deviation
MSD: mean squared deviation
BBD: Box-Behnken design
