# Supplementary material for: Application of a strategy based on metabolomics guided promoting blood circulation bioactivity compounds screening of vinegar
Source: Chem Cent J. 2017 May 8;11:38. doi: 10.1186/s13065-017-0265-5 (PMC5422338; doi:10.1186/s13065-017-0265-5)
Supplement: Supplementary file 1 — Additional file 1: Table S1. The content of TMPZ in RV and WV. Table S2. The peak area and the relative peak area value of four potential biomarkers in different aging period. Table S3. The levels and factors investigated in BBD. Figure S1. HPLC chromatogram of TMPZ. Figure S2. The results of bioactivity screening. Figure S3. Diagnostic efficacy evaluation using ROC curves of the four potential biomarker metabolites in two different vinegar. Figure S4. Trends of time-series analysis graphs of four potential biomarkers. (A) TMPZ (MAPE: 2.05853, MAD: 1.67627, fitted curve: Yt = 60.81+5.089xt); (B) Dihydroergotamine (MAPE: 1.63096, MAD: 0.15345, fitted curve: Yt = 6.726+0.7121xt); (C) Harmine (MAPE: 1.72704, MAD: 0.01711, fitted curve: Yt = 0.7764+0.05780xt); (D) 1,2,3,4-tetrahydroharmine (MAPE: 3.76071, MAD: 0.04998, fitted curve: Yt = 0.9695+0.0910xt). Figure S5. Response surfaces estimated from the full factorial design for the content of total alkaloids. [file 13065_2017_265_MOESM1_ESM.docx]

**Application of a strategy based on metabolomics guided promoting blood circulation bioactivity compounds screening of vinegar**

**Zhangchi Ning^§1^, Zhenli Liu^§1^, Zhiqian Song^1^, Chun Wang^1^, Yuanyan Liu^2^*, Jiahe Gan^1^, Xinling Ma^1^, Aiping Lu^3^***

**Zhangchi Ning:** [**yizhangyichi1573@sina.com**](mailto:yizhangyichi1573@sina.com)**; Zhenli Liu:** [**Zhenli_liu@sina.com**](mailto:Zhenli_liu@sina.com)**; Zhiqian Song:** [**szy0801_2001@126.com**](mailto:szy0801_2001@126.com)**; Chun Wangle:** [**chuner-2006@hotmail.com**](mailto:chuner-2006@hotmail.com)**; Yuanyan Liu:** [**yyliu_1980@163.com**](mailto:yyliu_1980@163.com)**; Jiahe Gan:** [**175185723@qq.com**](mailto:175185723@qq.com)**; Xinling Ma:** [**397559423@qq.com**](mailto:397559423@qq.com)**; Aiping Lu:** [**aipinglu@hkbu.edu.hk**](mailto:aipinglu@hkbu.edu.hk)**.**

**Affiliation**

^1^Institute of Basic Theory, China Academy of Chinese Medical Sciences, Beijing, China

^2^ School of Chinese Materia Medica, Beijing University of Chinese Medicine, Beijing, China

^3^School of Chinese Medicine, Hong Kong Baptist University, Hongkong, China.

^§^ These authors contributed equally to this work.

* Corresponding author: Prof. Aiping Lu, School of Chinese Medicine, Hong Kong Baptist University, Hong Kong SAR 00825, China. Tel.: (852) 3411 2457, Fax: (852) 3411 2461

*E-mail address:* aipinglu@hkbu.edu.hk(A.P. Lu).

Dr. Yuanyan Liu, School of Chinese Materia Medica, Beijing University of Chinese Medicine, Beijing 100029, China. Tel: +86 10 84738658, Fax: +86 10 84738611.

*E-mail address*: yyliu_1980@163.com (Y.Y. Liu)

**Contents**

**Table S1.** The content of TMPZ in RV and WV

**Table S2.** The peak area and the relative peak area value of four potential biomarkers in different aging period

**Table S3.** The levels and factors investigated in BBD

**Fig. S1.** HPLC chromatogram of TMPZ.

**Fig. S2.** The results of bioactivity screening.

**Fig. S3.** Diagnostic efficacy evaluation using ROC curves of the four potential biomarker metabolites in two different vinegar

**Fig. S4.** Trends of time-series analysis graphs of four potential biomarkers. (A) TMPZ (MAPE: 2.05853, MAD: 1.67627, fitted curve: Yt=60.81＋5.089xt); (B) Dihydroergotamine (MAPE: 1.63096, MAD: 0.15345, fitted curve: Yt=6.726＋0.7121xt); (C) Harmine (MAPE: 1.72704, MAD: 0.01711, fitted curve: Yt=0.7764＋0.05780xt); (D) 1,2,3,4-tetrahydroharmine (MAPE: 3.76071, MAD: 0.04998, fitted curve: Yt=0.9695＋0.0910xt).

**Fig. S5.** Response surfaces estimated from the full factorial design for the content of total alkaloids.

Table S1. The content of TMPZ in RV and WV

|  | No. | Aging time（Month） | Batch No. | Content (mg/L) |
| --- | --- | --- | --- | --- |
| WV | 1 | 11 | 20120817 | \ |
|  | 2 | 8 | 20121124 | \ |
|  | 3 | 18 | 20120121 | \ |
| RV | 4 | 1 | 20130623 | 10.1 |
|  | 5 | 4 | 20130323 | 22.4 |
|  | 6 | 5 | 20130217 | 32.0 |
|  | 7 | 7 | 20121226 | 33.9 |
|  | 8 | 14 | 20120506 | 45.6 |
|  | 13 | 20 | 20111131 | 101.9 |
|  | 11 | 30 | 20120105 | 109.5 |

Table S2. The peak area and the relative peak area value of four potential biomarkers in different Aging period

| Aging period  （month） | TMPZ | | 1,2,3,4-tetraharmine | | Harmine | | dihydroergotamine | |
| --- | --- | --- | --- | --- | --- | --- | --- | --- |
|  | Peak area | Relative peak area | Peak area | Relative peak area | Peak area | Relative peak area | Peak area | Relative peak area |
| 1 | 33414087 | 66.15 | 553405 | 1.10 | 414807 | 0.82 | 3714956 | 7.35 |
| 4 | 36573219 | 72.41 | 587654 | 1.16 | 464324 | 0.92 | 4230944 | 8.38 |
| 5 | 37678998 | 74.60 | 595422 | 1.13 | 465323 | 0.92 | 4276756 | 8.37 |
| 7 | 39242322 | 77.69 | 634533 | 1.26 | 496472 | 0.98 | 4654364 | 9.21 |
| 14 | 45324323 | 89.73 | 764321 | 1.51 | 547433 | 1.08 | 5263544 | 10.42 |
| 20 | 46493044 | 92.04 | 785323 | 1.55 | 574535 | 1.14 | 5644354 | 11.17 |
| 30 | 48242323 | 95.51 | 794332 | 1.57 | 589765 | 1.17 | 5867638 | 11.62 |

Table S3. The levels and factors investigated in BBD

|  | Factors | Normal | -1 level | +1 level |
| --- | --- | --- | --- | --- |
| A | the elution solvent | 50 % ethanol containing 5M ammonia aqueous | 25 % ethanol containing 5M ammonia aqueous | 75 % ethanol containing 5M ammonia aqueous |
| B | the volume of vinegar | 500 mL | 250 mL | 750 mL |
| C | the volume of resin | 500 mL | 250 mL | 750 mL |
| D | elution rate | 2 BV/h | 1 BV/h | 3 BV/h |


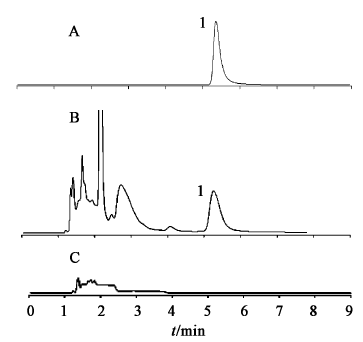


Fig. S1. HPLC chromatogram of TMPZ. (A)and sample RV (B), WV (C)


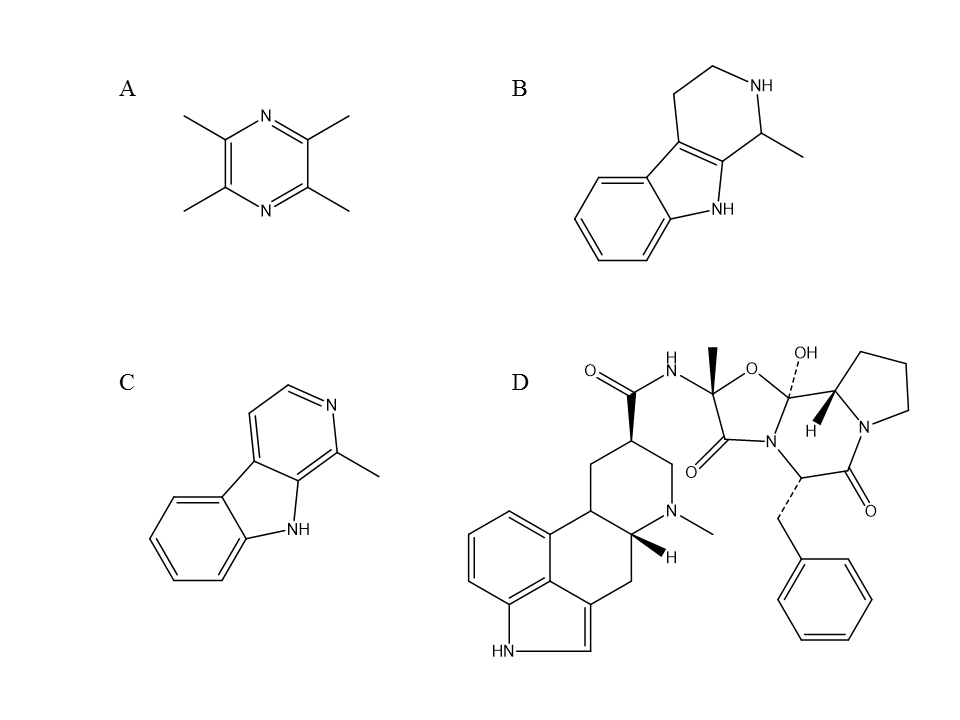


Fig. S2. The results of bioactivity screening. A. TMPZ; B. 1, 2, 3, 4-tetrahydroharmine; C. Harmine; D. Dihydroergotamine


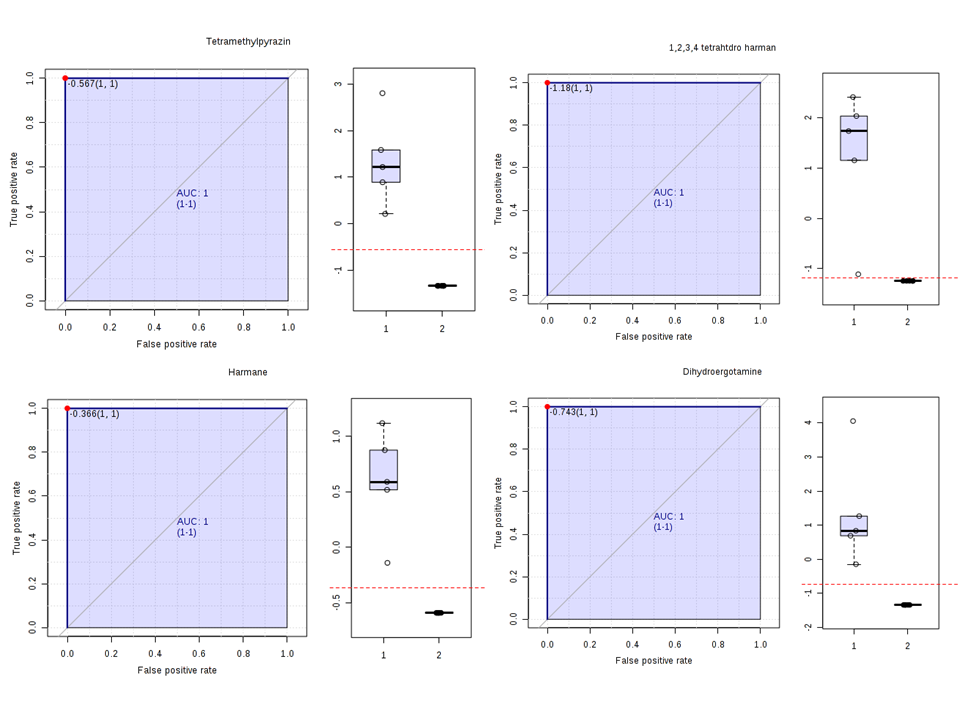


Fig. S3. Diagnostic efficacy evaluation using ROC curves of the four potential biomarker metabolites in two different vinegar


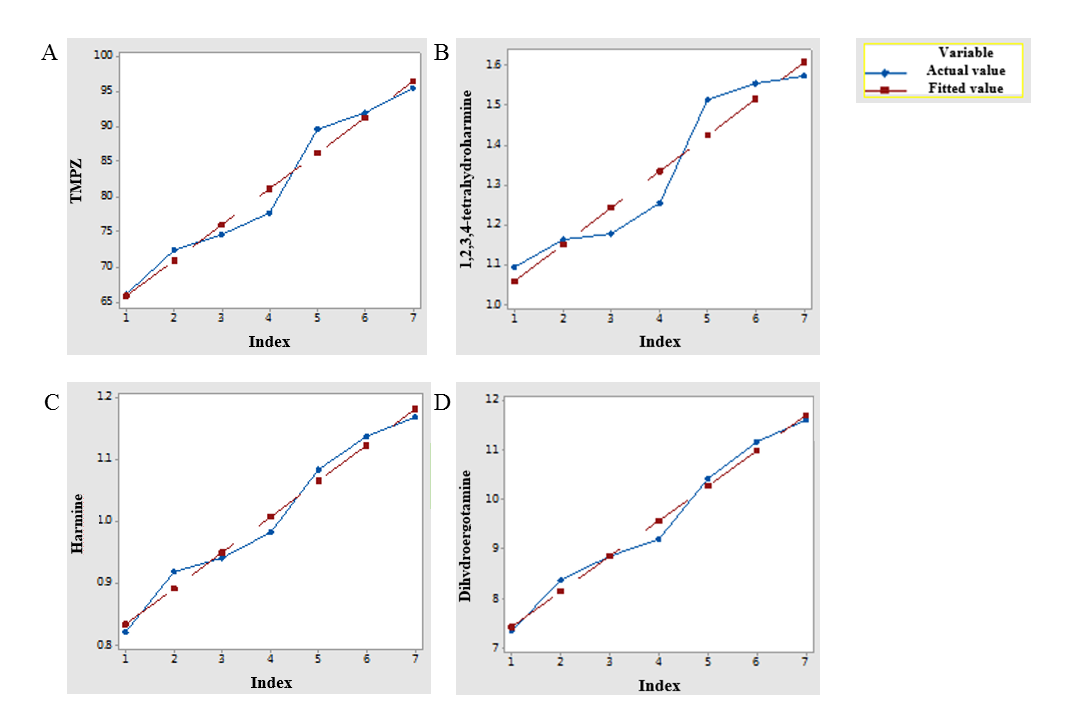


Fig. S4. Trends of time-series analysis graphs of four potential biomarkers.


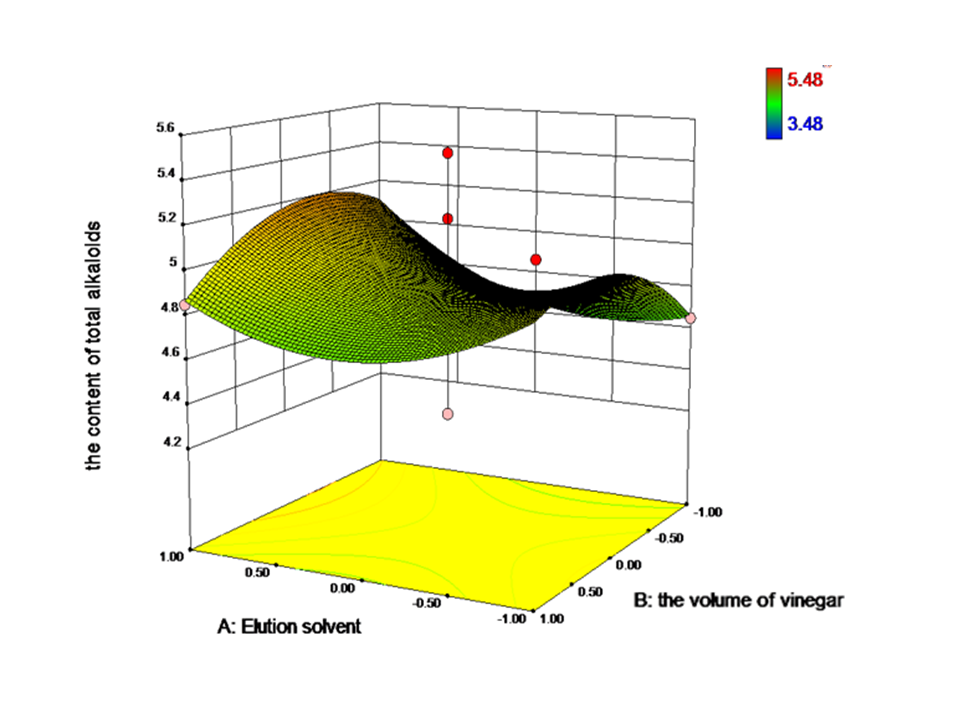


Fig. S5. Response surfaces estimated from the full factorial design for the content of total alkaloids.
